# Pragmatic Markers in the Management of Asthma: A Real-World-Based Approach

**DOI:** 10.3390/children7050048

**Published:** 2020-05-18

**Authors:** Giorgio Ciprandi, Gian Luigi Marseglia, Fabio Luigi Massimo Ricciardolo, Maria Angela Tosca

**Affiliations:** 1 Allergy Clinic, Casa di Cura Villa Montallegro, Via P. Boselli 5, 16146 Genoa, Italy; 2 Pediatric Clinic, Department of Pediatrics, Fondazione IRCCS Policlinico San Matteo, University of Pavia, 27100 Pavia, Italy; gl.marseglia@smatteo.pv.it; 3 Department of Clinical and Biological Sciences, University of Turin, San Luigi Gonzaga University Hospital, 10043 Turin, Italy; fabioluigimassimo.ricciardolo@unito.it; 4 Pediatric Allergy Center, Istituto Giannina Gaslini, 16100 Genoa, Italy; mariangelatosca@gaslini.org

**Keywords:** asthma, biomarkers, phenotype, endotype, personalized therapy

## Abstract

Bronchial hyperreactivity, reversible airflow limitation and chronic airway inflammation characterize asthma pathophysiology. Personalized medicine, i.e., a tailored management approach, is appropriate for asthma management and is based on the identification of peculiar phenotypes and endotypes. Biomarkers are necessary for defining phenotypes and endotypes. Several biomarkers have been described in asthma, but most of them are experimental and/or not commonly available. The current paper will, therefore, present pragmatic biomarkers useful for asthma management that are available in daily clinical practice. In this regard, eosinophil assessment and serum allergen-specific IgE assay are the most reliable biomarkers. Lung function, mainly concerning forced expiratory flow at 25-755 of vital capacity (FEF_25-75_), and nasal cytology may be envisaged as ancillary biomarkers in asthma management. In conclusion, biomarkers have clinical relevance in asthma concerning both the endotype definition and the personalization of the therapy.

## 1. Introduction

Asthma represents a common disease in childhood and adolescence because of the high prevalence (about 5%–10%), chronic nature, the potentially severe symptoms, and the relevant burden on healthcare resources. The definition of asthma, provided by the Global Initiative for Asthma (GINA) guidelines, describes it as a heterogeneous disease [1]. Different phenotypes and endotypes characterize distinct clinical characteristics and specific pathophysiological mechanisms [2,3]. Airway inflammation and hyperresponsiveness are the mainstay of asthma and govern the clinical scenario. In this regard, type 2 inflammation is the predominant phenotype in asthmatic children and adolescents [4]. Type 2 inflammation occurs when at least one factor is decisive, including blood eosinophils > 300 cells/µL, fractioned exhaled nitric oxide (FeNO) > 35 ppb (in children), total IgE > 100 IU/mL, and allergy [5,6]. Type 2 airway inflammation is characterized by an eosinophilic infiltrate closely associated with asthma exacerbations [7,8]. Therefore, abating eosinophilic inflammation could be an ideal therapeutic strategy for asthmatic patients. Personalized medicine is a tailored treatment strategy, taking into account the phenotype/endotype of the single subject to identify the more appropriate individual treatment [9,10]. To this purpose, the precision medicine approach is mandatory and consists of a thorough workup of asthmatic patients to precisely define the individual pheno/endotype [11]. Endotyping requires the availability of reliable markers able to identify the immunopathological characteristics that are specific in every subject [12,13]. 

On the other hand, the control of asthma represents the goal in the management of asthmatic patients [14]. GINA guidelines identify three levels of asthma control: well-controlled, partly controlled, and uncontrolled [1]. The assessment of asthma control considers some clinical variables, such as symptom frequency and severity [15]. Moreover, the asthma control also considers the future risk of adverse outcomes, including ≥1 exacerbation in the past year, poor adherence, incorrect inhaler technique, impaired lung function, smoking, and eosinophilia [1]. It is well known that uncontrolled asthma is a relevant risk factor for severe asthma exacerbation, impairs the quality of life of children, adolescents, and also their parents, and increases healthcare costs [15]. A control-based approach should be, therefore, preferred to manage asthmatic children and adolescents. 

Notably, the availability of new treatments, namely biologics, has changed the prescriptive attitude [16]. As a consequence, asthma management should include asthma control assessment and pheno/endotyping. However, there are very few available markers in clinical practice [17]. Many of the proposed biomarkers are still experimental and cannot be used in daily activity. Recently, it has been proposed that blood eosinophils, serum allergen-specific IgE, and lung function can be pragmatic markers to personalize asthma management [18]. As new evidence has been provided in the last years, the present review updates the list of pragmatic markers, evaluated in the real-world setting, that may be useful in the care of asthmatic children and adolescents. The real-world setting concerns the medical experience occurring in clinical practice, such as considering all patients examined. In particular, the information derived from a randomized controlled trial that involves selected patient populations rarely mirrors the real situation occurring in clinical practice [19]. We will, therefore, present pragmatic biomarkers useful for asthma management that are available in daily clinical practice.

## 2. Clinical Markers

In addition to conventional clinical variables, including symptoms, allergy, infections, and response to medications, some other parameters could be considered for integration into the assessment of asthma control.

### 2.1. Visual Analog Scale (VAS)

The perception of symptoms and drug use, measured by the visual analog scale (VAS), could be recognized as a very useful, simple, quick, and handy tool in clinical practice [20]. Formerly, a real-life pediatric study has demonstrated that the perception of breathlessness, assessed by VAS, was related with forced expiratory volume at 1 s (FEV_1_) and, in particular, a VAS value of 6 was found to be a reliable cutoff (area under the curve = 0.83; Odds Ratio = 9.4) for discriminating children with bronchial obstruction [21]. The clinical relevance of this study was that the VAS assessment, everywhere feasible, may quickly provide an approximative idea of lung function and suggest the necessary measures. The pragmatic proposal was a four-colored scale: (i) green zone (children with VAS value between 8 and 10) means that you do not worry; (ii) yellow zone (VAS 6–8) means to see and wait; (iii) orange zone (VAS 4–6) means to refer to the pediatrician and consider spirometry; and (iv) red zone (VAS < 4) means to treat with relievers [21] immediately. A second study evidenced that VAS could also be considered a primary tool, for example, at home or in the pediatrician’s office, to predict the bronchial reversibility [22]. A further study suggested that VAS could be used to estimate the patient’s perception of short-acting β2-agonists used in clinical practice [23]. The awareness of relievers use could, therefore, improve the belief of asthma severity and potentially improve the self-management. More recently, it has been reported that the VAS assessment of asthma symptoms was linked with the asthma control grade [24]. Asthmatic children with uncontrolled, but also with partly controlled, asthma had the lowest VAS scores.

These outcomes suggest, therefore, that VAS assessment of asthma symptoms could be used in the management of asthmatic children and adolescents, even though VAS concerns the subjective perception of symptoms and should be integrated by objective measurements.

### 2.2. Emotional Aspects

There is consensus that the emotional disorders, namely anxiety and depression, frequently affect adolescents who have asthma [25] and are common also in their parents [26]. A recent systematic review and meta-analysis concluded that caregivers of asthmatic children are more anxious and depressed than caregivers of healthy children [27]. Consequently, emotional disorders of the parents and anxiety and/or depression in their asthmatic children may affect asthma outcomes [28]. Parental emotional problems, mainly maternal, worsen the asthma severity and control, and increase asthma medication use in their children [29,30,31]. Moreover, adolescence is a critical age from an emotional point of view, as the adolescent is defining his/her identity and personality, maturing into a person and experiencing a range of new emotions [32]. Asthmatic adolescents have a weak acceptance of the asthma diagnosis, underestimate symptoms, have scarce compliance and adherence to the prescribed treatment, and self-management of asthma, mainly concerning the decision to take reliever drugs, is inadequate [33,34]. As a consequence, asthma control becomes a complicated task. In this regard, a real-world study conducted in asthmatic adults showed that anxiety and depression were common and significantly associated with uncontrolled asthma [35]. These outcomes were consistent with a recent real-world study showing that children and adolescents with uncontrolled and partly controlled asthma experienced more frequent anxiety and depression than their well-controlled peers [24]. A further study reported that anxiety and depression were common in asthmatic adolescents and their parents, mainly in their mothers [36]. Mostly, emotional disorders significantly affected asthma control, so that only 29% of adolescents had well-controlled asthma and, consistently, the lowest rate of emotional problems. Moreover, maternal anxiety was frequent in adolescents with uncontrolled asthma. A longitudinal study demonstrated that the standard asthma treatment improved the asthma control but did not affect the emotional pattern in their parents [37].

Therefore, these studies suggest that the assessment of emotional issues should be considered in the management of asthmatic children, adolescents, and their caregivers. However, confounding aspects, including literacy and socio-economic status, should be considered.

### 2.3. Type 2 Inflammation

Asthma in children and adolescents is predominantly characterized by type 2 inflammation, mainly by the allergic phenotype. Therefore, the assessment of type 2 markers is useful in asthma management. The primarily available markers for type 2 inflammation are serum IgE, blood eosinophils, FeNO, and periostin.

Total serum IgE has no clinical value in monitoring asthma control as there is evidence that it does not correlate with asthma control grade in follow-up studies [24,38,39]. However, a study provided conflicting results [40]. On the other hand, total IgE evaluation is required for prescribing and titrating omalizumab: adult and adolescent patients should have ≥76 IU/mL and children >200 IU/mL to be included in the treatment.

Allergen-specific IgE assessment is useful for phenotyping asthma, such as defining allergic asthma. The presence of sensitization, such as the production of allergen-specific IgE, allows for discriminating allergic asthma from non-allergic asthma [17]. In other words, the documentation of sensitization is essential to phenotype a patient and, if indicated, to identify the more appropriate biologic [9,10]. In particular, it is essential to distinguish allergic asthma and eosinophilic asthma, as different pathways are involved [11,12,13].

FeNO has been proposed as a reliable marker to measure eosinophilic airway inflammation. A longitudinal study first showed its utility to predict and diagnose poorly controlled asthma [41]. FeNO forecasted loss of control with a positive predictive value between 80% and 90%. These data showed that an absolute FeNO value of ≥15 ppb or 60% over baseline was a useful inception for ongoing airway inflammation detection and positively predicts breakthrough symptom arrival.

Several studies showed an increase of FeNO in asthma, further heightening when asthma control declines or when exacerbation occurs [42]. During corticosteroid therapy, FeNO changes come before symptoms, FEV_1_, and sputum eosinophilia improvement. Thus, FeNO can be considered a sensitive predictor of loss of asthma control [43]. Another study concluded that FeNO value > 300% of predicted identifies subjects at risk of excessive use of rescue medication and needing oral corticosteroids within one year [44]. However, a recent pediatric real-world study showed that FeNO did not discriminate children based on asthma control [24]. Therefore, FeNO assessment could have some clinical relevance, but its use is still controversial.

Blood eosinophils steadily correlate with sputum recovered from bronchial lavage or biopsic eosinophils [11]. Therefore, blood eosinophil count is commonly used in clinical practice to assess type 2 inflammation [45,46]. Moreover, blood eosinophil count is a predictive parameter that identifies responders to biologics [47,48,49]. Therefore, blood eosinophil count should be considered a useful marker in the management of asthmatic children and adolescents.

Serum periostin: there are conflicting findings concerning the real value of periostin as a reliable asthma biomarker. Wagener showed that serum periostin was not related to sputum eosinophils [50]. Mansur demonstrated that FeNO had a stronger correlation with asthma exacerbations than blood eosinophils or periostin [51]. Consistently, a real-world pediatric study demonstrated that serum periostin was not associated with the asthma control grade [52]. El Basha reported conflicting results showing that serum periostin was linked with severe asthma and asthma exacerbation [53]. From a practical point of view, the use of serum periostin is still experimental, and its assessment should not be performed in clinical practice.

### 2.4. Lung Function

Lung function evaluation, mainly concerning FEV_1_ and the rate between FEV_1_ and forced vital capacity (FVC), is mandatory for asthma diagnosis and monitoring. In particular, FEV_1_ has always been considered the gold standard in the interpretation of spirometry in asthmatic patients [54]. However, FEV_1_ values may frequently be in the normal range when evaluating children and adolescents with asthma [55]. Therefore, there is a growing interest in the definition of the practical role exerted by the forced expiratory flow between 25% and 75% of forced vital capacity (FEF_25-75_). FEF_25-75_ may have clinical relevance, especially when FEV_1_ values are normal [56]. Indeed, it has been proposed that reduced FEF_25-75_ value might precede FEV_1_ impairment, therefore indicating early disease and poor prognosis [57]. There is a body of evidence that suggests FEF_25-75_ as a reliable marker to monitor asthmatic patients [58,59]. Moreover, there is evidence that FEF_25-75_ is significantly associated with the asthma control grade [60,61,62,63]. Therefore, FEF_25-75_ should be included in the spirometry parameters evaluated in daily practice.

### 2.5. Asthma Control Questionnaire (ACT)

Asthma control may also be assessed by the Asthma Control Test (ACT) questionnaire developed for use in clinical practice [64]. The ACT questionnaire consists of five questions with five possible responses, exploring the patient’s perception of his/her asthma control. The result could range between 0 and 25, where 25 is the optimal asthma control. Many studies confirmed that ACT correlated well with the asthma control grade [65,66,67]. A meta-analysis also provided convincing evidence concerning its reliability in assessing the asthma control grade [68]. Moreover, ACT can predict future asthma risks [69]. Interestingly, a pediatric version is also available, the children ACT (cACT), that provides fruitful information in asthma management [70]. A real-life study underlined its benefit in assessing asthmatic patients [71]. However, it has been reported that ACT scores did not correlate with the asthma control grade proposed by GINA guidelines [72]. This outcome was confirmed in a pediatric study showing that the frequencies of the two categorizing methods did not agree between them [24]. Therefore, both ways should be pursued in asthma control grading. ACT is a reliable tool to assess and monitor asthma control and provides quick and straightforward information about the patient’s perception of asthma control. On the other hand, ACT reflects the perception of asthma control and consequently could be influenced by confounding factors, including emotional issues.

## 3. Conclusions

**Figure 1:**
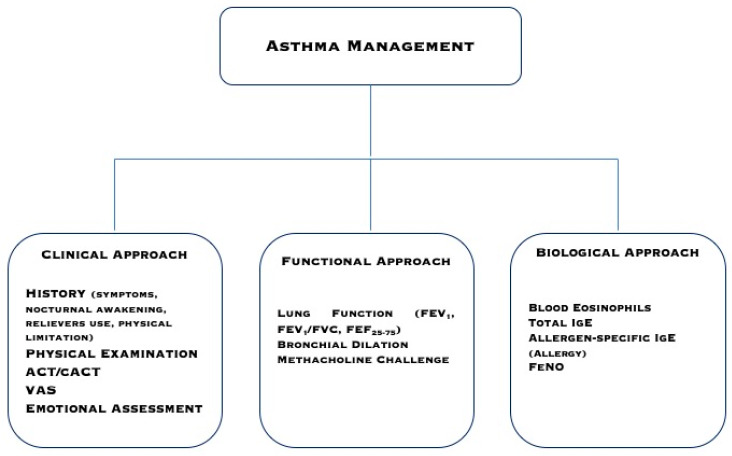
A pragmatic approach to manage asthmatic patients.

At present, very few inflammatory biomarkers are validated and reliable. In the common practice, such as in primary and secondary care levels, but also tertiary levels in some geographical areas, the use of biomarkers is minimal for financial reasons. We would like to propose a pragmatic workup using popular markers that are routinely evaluated in a real-life setting and are available everywhere (Figure 1). Blood eosinophils and allergen-specific IgE should be measured in all children and adolescents with asthma. They may give information about allergy presence, type 2-driven bronchial inflammation, asthma severity, and responsiveness to both steroids and allergen-immunotherapy (AIT). With these two simple biomarkers, it is possible to manage asthmatics, following the criteria of precision medicine, in every real-life setting. Blood eosinophils and allergen-specific IgE are fundamental to differentiate the allergic phenotype (both markers are positive) from the eosinophilic phenotype (absence of allergen-specific IgE). Phenotyping/endotyping is useful to identify the most appropriate therapy for every patient. On the other hand, other phenotypes and endotypes exist, including neutrophilic and non-allergic asthma. As a consequence, additional investigations may be required in some patients.

ACT, VAS, and emotional assessments are straightforward and quick tools that allow for obtaining relevant information. Spirometry is mandatory to diagnose asthma and correctly manage the patient. However, as FEV_1_ may be frequently normal and bronchodilation testing and the methacholine challenge are complex exams, FEF_25-75_ is a simple parameter that could give relevant information concerning early bronchial airflow limitation.

Therefore, it seems to be an acceptable pragmatic approach to consider these parameters as common markers. Therefore, the daily clinical practice suggests considering a simple pathway that should be based on symptom history, functional assessment, response to conventional treatment, and measurement of basilar inflammatory biomarkers. However, further studies are needed to validate this integrated approach.

In conclusion, pragmatic markers have clinical relevance in asthma management concerning the assessment of asthma control, endotype definition, and personalization of the therapy.
